# Cost-Effectiveness of Initiating Pharmacological Treatment in Stage One Hypertension Based on 10-Year Cardiovascular Disease Risk: A Markov Modeling Study

**DOI:** 10.1161/HYPERTENSIONAHA.120.14913

**Published:** 2020-12-21

**Authors:** Margaret Constanti, Christopher N. Floyd, Mark Glover, Rebecca Boffa, Anthony S. Wierzbicki, Richard J. McManus

**Affiliations:** 1 From the National Guideline Centre (NGC), Regent’s Park, London (M.C., R.B.); 2 Department of Clinical Pharmacology, King’s College London, St Thomas’ Hospital Campus (C.N.F.); 3 MRC Clinician Scientist, Faculty of Medicine and Health Sciences, Queen’s Medical Centre, Nottingham (M.G.); 4 Department of Metabolic Medicine/Chemical Pathology, Guy’s and St Thomas’ Hospitals, London (A.S.W.); 5 Nuffield Department of Primary Care Health Sciences, University of Oxford (R.J.M.).

**Keywords:** blood pressure, cardiovascular diseases, economic model, primary prevention, quality-adjusted life-year

## Abstract

Antihypertensive drug treatment is cost-effective for adults at high risk of developing cardiovascular disease (CVD). However, the cost-effectiveness in people with stage 1 hypertension (140–159 mm Hg systolic blood pressure) at lower CVD risk remains unclear. The objective was to establish the 10-year CVD risk threshold where initiating antihypertensive drug treatment for primary prevention in adults, with stage 1 hypertension, becomes cost-effective. A lifetime horizon Markov model compared antihypertensive drug versus no treatment, using a UK National Health Service perspective. Analyses were conducted for groups ranging between 5% and 20% 10-year CVD risk. Health states included no CVD event, CVD and non-CVD death, and 6 nonfatal CVD morbidities. Interventions were compared using cost-per-quality-adjusted life-years. The base-case age was 60, with analyses repeated between ages 40 and 75. The model was run separately for men and women, and threshold CVD risk assessed against the minimum plausible risk for each group. Treatment was cost-effective at 10% CVD risk for both sexes (incremental cost-effectiveness ratio £10 017/quality-adjusted life-year [$14 542] men, £8635/QALY [$12 536] women) in the base-case. The result was robust in probabilistic and deterministic sensitivity analyses but was sensitive to treatment effects. Treatment was cost-effective for men regardless of age and women aged >60. Initiating treatment in stage 1 hypertension for people aged 60 is cost-effective regardless of 10-year CVD risk. For other age groups, it is also cost-effective to treat regardless of risk, except in younger women.

## Abstract 2

Supplemental Digital Content is available in the text.

Hypertension is one of the most important reversible risk factors for global morbidity and mortality.^1^ Pharmacological treatment of hypertension reduces all-cause mortality and incidence of cardiovascular disease (CVD) events, including heart attacks and strokes.^2^

Recommendations for antihypertensive drug treatment in England are based, wherever possible, on the cost-effectiveness of treatment, with a threshold of <£20 000/quality-adjusted life-year (≈$29 000/QALY).^3,4^ Previous UK National Institute for Health and Care Excellence (NICE) guidance for treatment initiation in those with stage 1 hypertension was consensus-based and suggested treatment for those aged under 80 with a 10-year CVD risk of 20% or greater.^5^

The systematic review comparing antihypertensive drug treatment at different blood pressure (BP) thresholds, undertaken as part of updating the NICE hypertension in adults guideline,^6^ showed a reduction in CVD events from antihypertensive treatment for people with stage 1 hypertension (clinic BP 140–159/90–99 mm Hg). However, uncertainty remained about the cost-effectiveness of treatment in this population because at lower absolute risk levels, the number needed to treat was higher, and the balance of benefits and risks varied with different CVD risk levels.

In providing evidence for the 2019 NICE hypertension guideline, this study aimed to establish the 10-year CVD risk level at which initiation of antihypertensive treatment in people with stage 1 hypertension was cost-effective.

## Methods

Data used in the study was sourced from the literature as cited.

A lifetime cost-utility analysis assessed the risk threshold at which antihypertensive treatment of stage 1 hypertension became cost-effective. A Markov model was developed for the National Health Service, comparing drug versus no drug treatment in people aged 40 and over. The model is briefly described below, with further technical details of the modeling available in the Data Supplement, and NICE website.^6^

### Base-Case and Comparators

The population had stage 1 primary hypertension without target organ damage, established CVD, renal disease, or diabetes.^7^ In the base-case, the model used a starting age of 60 for both sexes. Alternative starting ages were chosen to represent the treated population for whom data exist (40, 50, 60, 70, and 75) and were considered in sensitivity analyses for each CVD risk subgroup and sex.^6^

Antihypertensive drug treatment was compared with no antihypertensive drug treatment in four 10-year CVD risk subgroups: 5%, 10%, 15%, and 20%. Both treated and untreated groups were assumed to receive equal treatment regarding other CVD reduction strategies.

### Model Structure

The Markov model used a 1-year cycle length and calculated lifetime costs and QALYs for each comparator (Figure). Outcomes included death (either from CVD or non-CVD) and 6 types of nonfatal CVD events (Data Supplement).

**Figure.**
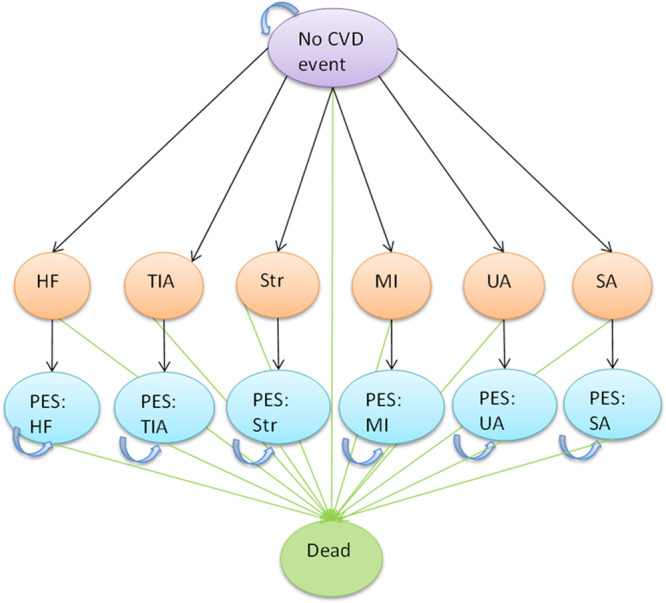
**Model structure.** CVD indicates cardiovascular disease; HF, heart failure; MI, myocardial infarction; SA, stable angina; TIA, transient ischemic attack; and UA, unstable angina.

As this was a primary prevention population, all subjects entered the model in the no CVD event state. Subsequent events occurred depending on risk. For each nonfatal CVD event, event and postevent states were used to apply different costs and utilities in the first and subsequent cycles (all 1 year). Repeat events were not explicitly modeled.

The model was run for a maximum of 60 cycles (to age 100), by which time most people in the model would have died.

### Model Inputs

A summary of the model inputs used in the base-case (primary) analysis is provided in Table 1, with further detail in the Data Supplement.

**Table 1.**
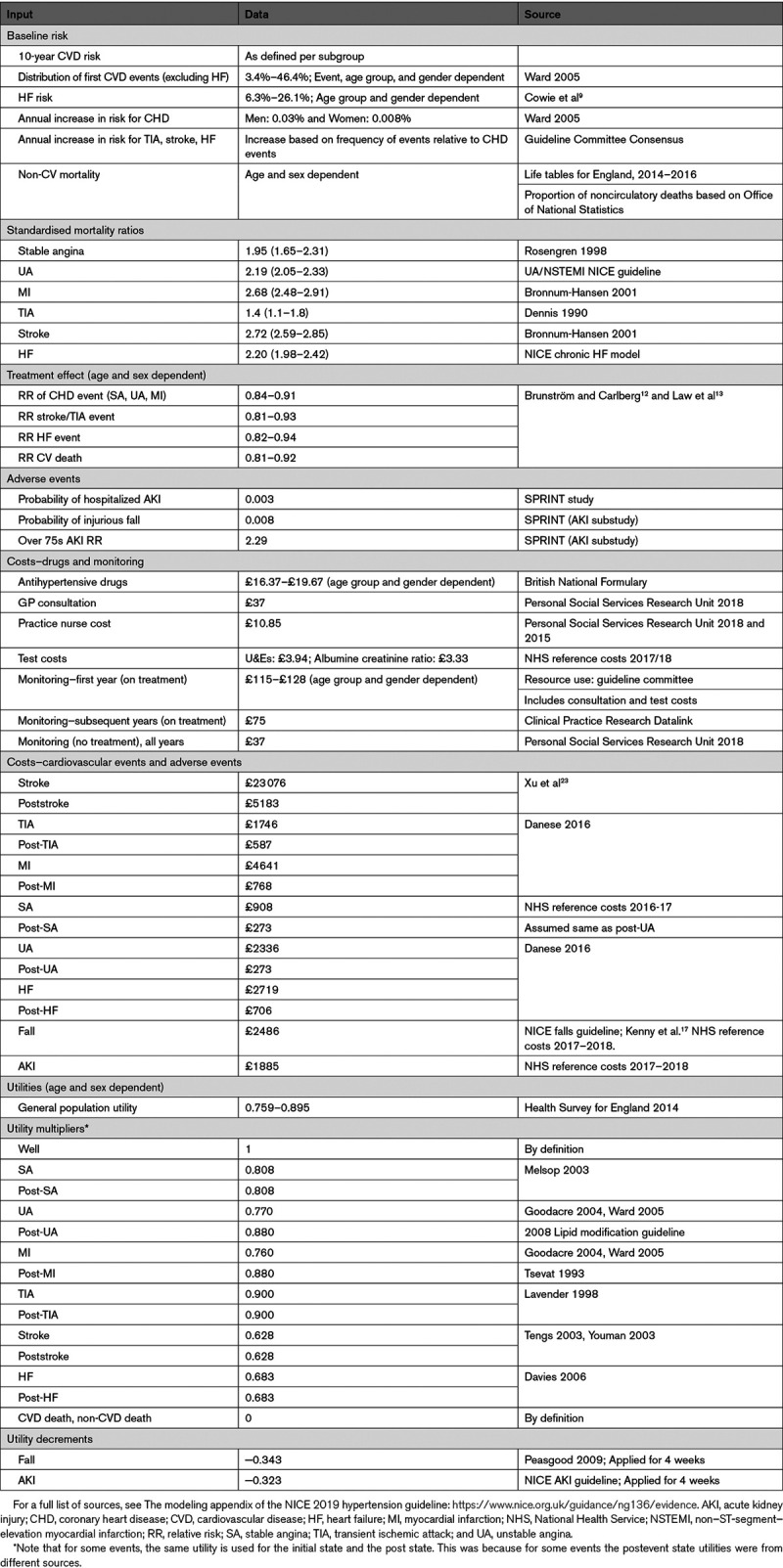
Summary of Base-Case Model Inputs

### Baseline Risks

Annual transition probabilities were calculated for each CVD event in the model,^8^ considering as follows:

- The 10-year CVD risk of the risk subgroup (5%, 10%, 15%, or 20%),
- The relative distribution of types of CVD event, ^9,10^
- How CVD risk changes over time.

Table S1 in the Data Supplement shows the relative distributions of first CVD events by age and sex.

The annual risk of a first CVD event increased by a fixed amount each year to account for increasing age. This was applied as an additive percentage, with risk calibrated so that the sum of the risk of a first event plus the annual increase in risk was equal to the 10-year risk when compounded over 10 years.^10^

### Mortality

Non-CVD mortality was estimated using lifetables, applying the proportion of noncirculatory deaths to the mortality rates by age and sex.^11^ Mortality after a CVD event was based on standardized mortality ratios (Table 1).

### Relative Treatment Effects

Treatment effects were taken from the only systematic review of randomised controlled trials identified from the guideline clinical review of stage 1 hypertension (Brunström and Carlberg),^12^ adjusted using relative age transformations calculated from a meta-analysis of treatment effects (Law et al^13^; Tables S2 and S4). Relative risk was assumed constant across all risk subgroups, although absolute treatment benefit still varied with baseline risk.

### Adverse Events

Adverse events were modeled using data from the SPRINT (Systolic Blood Pressure Intervention Trial; Data Supplement).^14–17^

### Utilities

Baseline quality of life was based on general population estimates stratified by age and sex.^18^ Quality of life decrements associated with CVD events and adverse events were taken from the literature, with CVD event utilities applied multiplicatively to the general population weights, and adverse event decrements applied as disutilities.^8,19,20^

### Resource Use and Costs

The cost of antihypertensive drug treatment was applied to everyone alive on treatment and to all following a nonfatal CVD event. Drug costs were taken from the British National Formulary for the most commonly used drug in each class, taking into account variation in the number of drugs prescribed (Table S3).^21,22^ For detailed monitoring costs, see Data Supplement.^23–25^

The costs of CVD events were identified from the literature (Table 1) and inflated to 2016/17 prices.^26^

### Analysis

A lifetime cost-utility analysis was undertaken using a Markov model. QALYs and costs from a current UK National Health Service and Personal Social Services perspective were considered, both discounted at 3.5% per annum in line with the NICE reference case.^3^ The model was constructed in Microsoft Excel 2010 and evaluated by cohort simulation.

QALYs and costs were half-cycle corrected, reflecting the assumption that people will transition between states on average halfway through a cycle. An incremental cost-effectiveness ratio (ICER) was calculated as the difference in costs divided by the difference in QALYs between the 2 strategies, with results presented as cost-per-QALY-gained. The cost-effectiveness of antihypertensive treatment was considered in relation to the NICE threshold of £20 000 per-QALY-gained.

Number needed to treat was calculated using the crude average of the relative risk across all events for men and women in each age group.

### Sensitivity Analyses

Probabilistic sensitivity analysis was undertaken to assess parameter uncertainty, with distributions attached to inputs in the model where possible (Data Supplement).

Deterministic sensitivity analyses conducted including running the model for alternative age groups (probabilistic) and testing differential treatment durations in the no treatment group (probabilistic; Table S7).

Other notable (probabilistic) sensitivity analyses around treatment effect included:

- Using different treatment effect estimates (Table S8),^13^
- Adjusting the base-case treatment effect to reflect treatment effect from using >1 drug (Tables S9 and S10).^12^

Various deterministic sensitivity analyses further tested the robustness of model inputs (Data Supplement).

### Threshold Analysis

For each age group and sex, the exact risk level (or threshold) at which treatment became cost-effective was explored.^27,28^

## Results

### Base-Case

For men and women aged 60, antihypertensive treatment in stage 1 hypertension was associated with improved QALYs but higher costs (Table 2) for all risk thresholds. At 10% risk, antihypertensive treatment was cost-effective for both men (£10 017 [$14 542]/QALY) and women (£8635 [$12 536]/QALY) with at least 85% probability (Figures S1 through S4). The threshold analysis found antihypertensive treatment became cost-effective (at £20 000 [$29 035]/QALY) at around 5% for both men and women, with significant uncertainty (50% and 51% probability cost-effective, respectively).

**Table 2.**
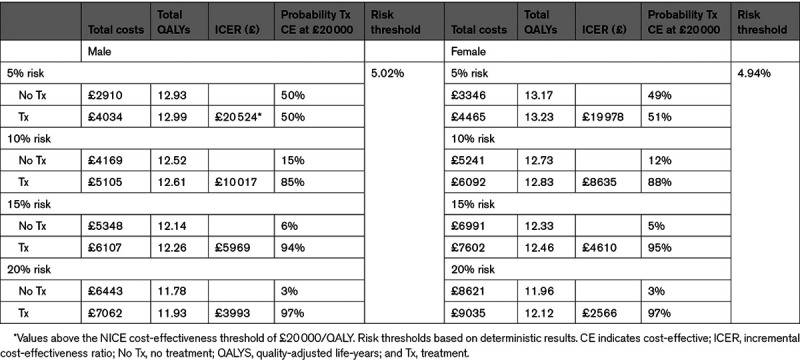
Base-Case Results for Individuals Aged 60 Years

### Results From Other Age Groups (Probabilistic)

A similar pattern was seen in other age groups: as risk increased, there were smaller incremental costs and higher incremental QALYs, leading to smaller ICERs (Table 3).

**Table 3.**
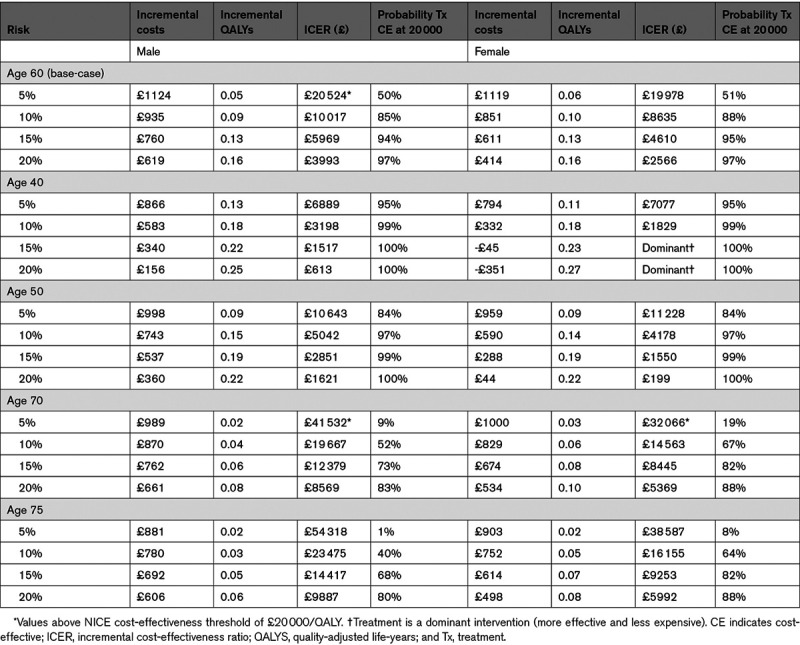
Results for Other Age Subgroups

In all male age groups, and for females age 60 and older, treatment regardless of CVD risk was cost-effective (Table 4).

**Table 4.**
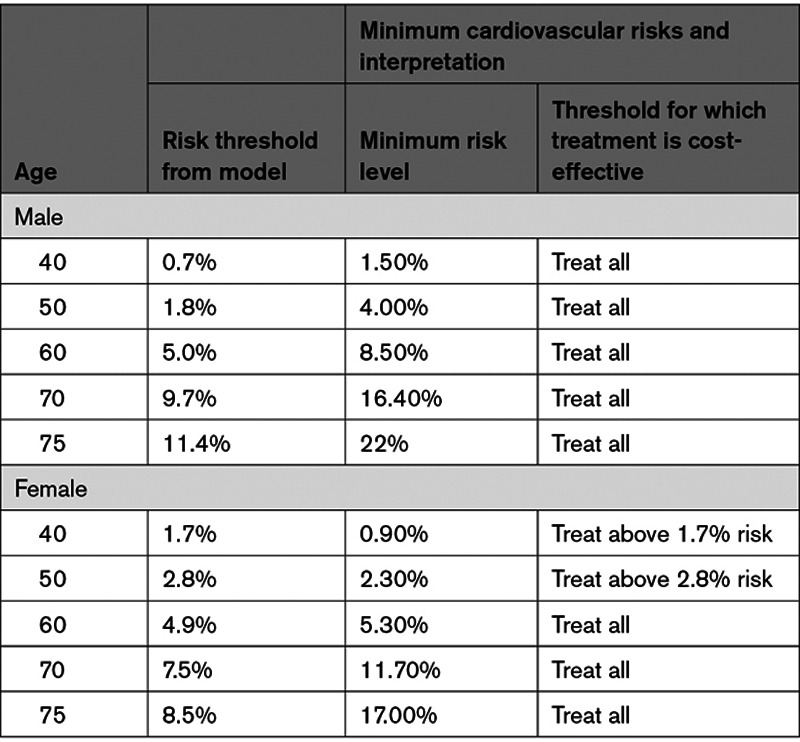
Model Interpretation Based on Cardiovascular Risk

The number needed to treat to avoid one CVD event over 10 years varied from 5 to 79 for men and 7 to 136 for women (Table S6).

### Sensitivity Analyses

The results of the model were sensitive to treatment effects. Treating hypertension in lower-risk groups, for those age 60, became more cost-effective as the effect size increased. Similar (often dominant) results were found in older age groups (Tables S12 and S13).

The differential treatment duration analysis (Table S11) showed it was cost-effective to treat all people with stage 1 hypertension aged 60, regardless of how soon they became eligible for treatment from other reasons (Data Supplement for discussion of other age groups).

Varying inputs that favored treatment or reduced costs led to lower ICERs, for example, more favorable treatment effect, or having no serious adverse events (Table S14). Conversely, as expected, varying inputs that would bias against treatment led to increased ICERs.

## Discussion

### Summary of Main Results

This economic model has shown it is generally cost-effective to treat stage 1 hypertension with antihypertensive drugs in all men (regardless of age) and all women (aged 60 or over). In younger women, the population risk profile was such that some would not reach the minimum threshold above which treatment was cost-effective, suggesting individual risk calculation might be appropriate.

Results were generally robust to sensitivity analyses. The differential treatment duration analysis for the base-case age showed it remained cost-effective to treat all those aged 60 with stage 1 hypertension. The cost-effectiveness of treatment for younger men depended on whether they would become eligible for antihypertensive treatment within around 20 years. It was still not cost-effective to treat all younger women, regardless of durations tested. The model was most sensitive to treatment effect, where more favorable treatment effects resulted in ICERs for all age/sex/risk groups that were either very low or treatment was dominant.

### Comparison With Literature

To our knowledge, this is the first economic evaluation of initiating treatment in different CVD risk groups in this patient population.

The model was based on a recent meta-analysis of the efficacy of antihypertensive drug treatment in primary prevention by BP group, of which stage 1 hypertension was one.^12^ The total included population had an average age of 63 and average systolic BP 154 mm Hg. For those only with stage 1 hypertension, the patient characteristics were not summarized, however, as the majority of trials labeled as primary prevention included some populations with comorbidities (eg, diabetes), the average CVD risk could be higher than a truly low-risk population.

Results for the lower-risk subgroups in the model need to be interpreted with caution. An observational study of antihypertensive treatment in a moderate risk group, using UK primary care data over a median follow-up period of 5.8 years, found no benefit of antihypertensive treatment for mortality (hazard ratio, 1.02 [0.88–1.17]) or CVD events (hazard ratio, 1.09 [0.95–1.25]) and an increased risk of adverse events.^29^

Other studies have used different methods to assess thresholds for initiating antihypertensive treatment. Another systematic review (Ettehad et al 2016) found a benefit for treatment on the basis of risk below 140/90 mm Hg.^30^ However, the review included populations with CVD, so outcomes were more representative of secondary rather than primary prevention.

The results for those receiving no treatment were validated using UK life tables and were consistent, suggesting that the assumptions used here were appropriate.^11^

### Limitations of the Model

The model was considered conservative for various reasons, including the structural assumption that people on no treatment would never commence antihypertensive treatment, unless they had a CVD event. This assumption was tested in sensitivity analyses, which did not change the conclusions in younger women but implied that risk assessment would be helpful in younger men who might become eligible for treatment for other reasons within 20 years. This reinforced the uncertainty around treatment thresholds for younger people, who are likely to have lower risk, and for whom 10-year risk calculators underestimate lifetime CVD risk.^31,32^ Furthermore, vascular damage in younger individuals may be preventable but irreversible later in life.^33^ Other possible benefits from taking antihypertensive treatment such as a possible reduction in dementia would also underestimate treatment benefit.^34,35^

The model was also conservative by not modeling repeat CVD events. This reduced complexity and data requirements. Health state costs that included future event costs were used wherever possible. However, if avoiding one event from treatment also avoided future events, it is likely that including repeat events would have made treatment more cost-effective.

Treatment effects used were conservative as they were mostly derived from studies of single drug interventions.^12^ Sensitivity analyses suggested more favorable treatment effects would make treatment more cost-effective in all groups. However, little data were available from truly lower-risk people with stage 1 hypertension, as such individuals are rarely included in RCTs.^29^ This reinforced the uncertainty about the most appropriate treatment threshold in younger-/lower-risk people due to the assumption of constant relative benefit across risk subgroups.^29^ However, the risk thresholds tested were close to the feasible risk levels, and so antihypertensive treatment is likely to be cost-effective for those with additional risk factors and stage 1 hypertension. As 87% of UK adults have at least one risk factor, and around half have 2 or more, on balance, it may be cost-effective to treat stage 1 hypertension in the majority of the population.^36^

The underlying risk used in the model is not affected by the calculation method and, therefore, would be generalizable to other settings, providing costs were similar. Specifically, the performance of the US atherosclerotic CVD risk calculator is similar to the UK QRISK2 calculator (used in this study)^37^ and would give equivalent outcomes.^38^ Similarly, using QRISK3, which predicts slightly lower risks for each age, would have little effect on the interpretation of the results.^39^

Another factor not considered was the variability in CVD risk over time, which was assumed to increase linearly, but might increase at a faster rate at certain time points, particularly in older people.^40^ This would increase the absolute benefit from treatment in those age groups. However, as older people have a much higher risk, treating older people regardless of risk was shown to be cost-effective.

A 1-year cycle length was chosen, and although a shorter cycle length can reduce the error of estimates produced, such benefits are modest with a lifetime horizon in a low-risk population, and bring significant computational burden. Furthermore, half-cycle correction was applied to improve precision.

In the model, utility decrements for adverse events were assumed to be additive. However, where lower quality of life is due to other comorbidities, this may have differential influences on quality of life from side effects.

Alternative modeling approaches such as microsimulation were considered. This could have improved the precision of population estimates. However, microsimulation would have added complexity and significant computational burden, with little clear benefit overall. In addition, the model structure was shared with the NICE lipid modification guideline,^8^ allowing for consistency in methods in CVD prevention models, facilitating consistency in interpretation.

Finally, adherence to treatment with antihypertensive drugs may differ between clinical trials and the real-world, leading to an overestimation of the treatment effect.^41^ However, there is insufficient published data to model the effect of adherence to antihypertensive drugs therapy on CVD events without excessive assumptions, which risk invalidating the model; this approach is consistent with other models.^8^ Any impact for suboptimal drug adherence is likely to be mitigated by the numerous conservative assumptions already described.

### Clinical Implications

The model supports a recommendation to lower the CVD risk threshold for antihypertensive treatment to 10% in stage 1 hypertension, with considerable uncertainty of benefit below that. This contrasts US and European guidance to treat all people with BP >140/90 mm Hg regardless of risk. This discrepancy can largely be explained by differing interpretation of the SPRINT results.^42^

Extrapolating the conclusions of this model to other health care systems is dependent on the costs of drug treatment, CVD risks, and adverse event rates in those settings. If treatment of CVD events was more expensive relative to antihypertensive treatment (as for example in the United States), antihypertensive treatment would be more cost-effective.^43^ Treatment of all stage 1 hypertension in high-cost settings may, therefore, be appropriate.

### Perspectives

This analysis found that treating people with stage 1 hypertension (without target organ damage, established CVD, renal disease, or diabetes) regardless of CVD risk was cost-effective across most age and sex subgroups, the exception being women under 60. The conclusions were sensitive to assumptions related to treatment efficacy and also differential initiation of treatment. Overall, antihypertensive treatment for individuals aged 60 or over with stage 1 hypertension is cost-effective in the UK National Health Service, but below this age, cost-effectiveness depends on CVD risk and how quickly an individual develops other indications for treatment. In these younger populations, additional potential benefits of antihypertensive treatment were not captured by the model and should be considered in making treatment decisions.^44^

## Acknowledgments

The guideline referred to in this article was produced by the National Guideline Centre for the National Institute for Health and Care Excellence (NICE). The views expressed in this article are those of the authors and not necessarily those of NICE. National Institute for Health and Care Excellence (2019), Hypertension in adults: Diagnosis and management. Available from https://www.nice.org.uk/guidance/ng136.

## Sources of Funding

The modeling exercise was undertaken as part of a National Institute for Health and Care Excellence (NICE) Guideline Update. NICE fund the salaries of M. Constanti and R. Boffa and part funded A.S. Wierzbicki in his role as guideline chair. R.J. McManus is an National Institute for Health Research Senior Investigator and receives part funding from the NIHR Oxford and Thames Valley Applied Research Consortium. C.N. Floyd is fully funded by the National Institute for Health Research M. Glover holds a Medical Research Council Clinician Scientist Fellowship (G1000861).

## Disclosures

R.J. McManus has received blood pressure (BP) monitors from Omron for research and is working with them to develop a telemonitoring system. He receives travel and accommodation expenses for occasional talks but does not personally accept honoraria or consultancy payments. The other authors report no conflicts.

## Supplementary Material



## Novelty and Significance

### What Is New?

- No adequately powered trials have assessed the effectiveness of treatment in stage 1 hypertension (blood pressure 140–159/90–99 mm Hg) in low-risk individuals (<10% 10-year cardiovascular disease risk).
- No previous study has assessed the most cost-effective risk threshold for starting treatment in people with stage 1 hypertension.
- This study used a modeling approach to consider long-term effectiveness of antihypertensive treatment in people with stage 1 hypertension at different levels of cardiovascular risk.

### What Is Relevant?

- It is cost-effective to treat most people with blood pressure >140/90 mm Hg regardless of risk. The exception being younger women (<60 years of age) where risk calculation is required.

### Summary

This newly developed economic model suggests it is cost-effective to treat all people aged 60 or more with stage 1 hypertension regardless of risk. There is more uncertainty in younger people, especially women, however, there may be other rationales for treatment in this group.
