# Supplementary figures and images for: Cost-Effectiveness of Initiating Pharmacological Treatment in Stage One Hypertension Based on 10-Year Cardiovascular Disease Risk: A Markov Modeling Study
Source: Hypertension. 2020 Dec 21;77(2):682–91. doi: 10.1161/HYPERTENSIONAHA.120.14913 (PMC7803450; doi:10.1161/HYPERTENSIONAHA.120.14913)

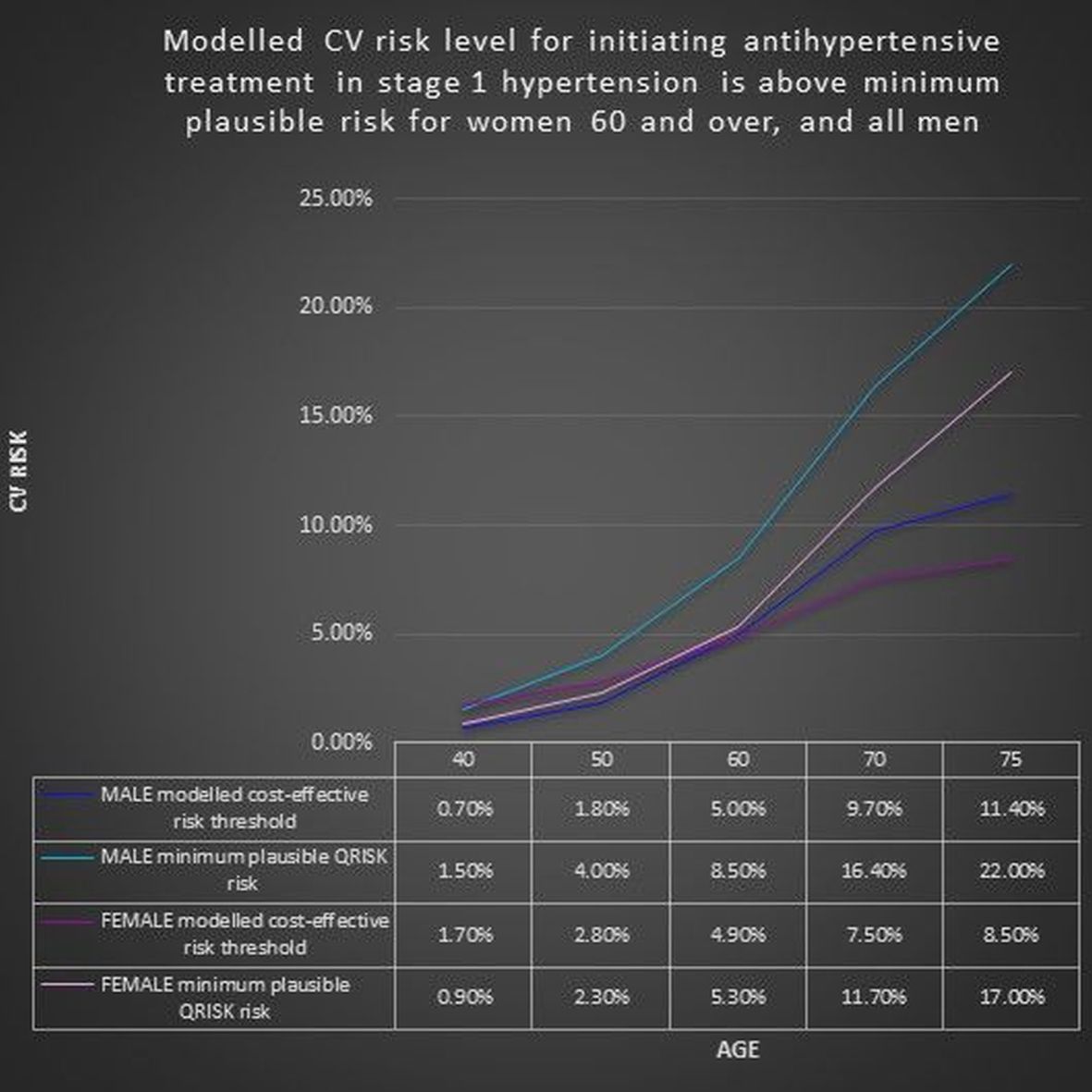

Supplement: Supplementary file 1 [file hyp-77-682-s001.jpg]
