# Supplementary material for: Cost-Effectiveness of Initiating Pharmacological Treatment in Stage One Hypertension Based on 10-Year Cardiovascular Disease Risk: A Markov Modeling Study
Source: Hypertension. 2020 Dec 21;77(2):682–91. doi: 10.1161/HYPERTENSIONAHA.120.14913 (PMC7803450; doi:10.1161/HYPERTENSIONAHA.120.14913)
Supplement: Supplementary file 2 [file hyp-77-682-s002.doc]

DATA SUPPLEMENT

**Cost-Effectiveness of Initiating Pharmacological Treatment in Stage One Hypertension Based on 10-Year Cardiovascular Disease Risk: A Markov Modelling Study**

Margaret Constanti^1^, Christopher N. Floyd^2^, Mark Glover^3^, Rebecca Boffa^1^, Anthony S. Wierzbicki*^4^, Richard J McManus.*^5^

1. National Guideline Centre (NGC), Regent’s Park, London NW1 4LE
2. Department of Clinical Pharmacology, King’s College London, St Thomas’ Hospital Campus, London SE1 7EH
3. MRC Clinician Scientist, Faculty of Medicine & Health Sciences, Queen's Medical Centre, Nottingham NG7 2UH
4. Department of Metabolic Medicine/Chemical Pathology, Guy’s & St Thomas’ Hospitals, London SE1 7EH
5. Nuffield Department of Primary Care Health Sciences, University of Oxford, Oxford OX2 6GG.

*Joint senior authors

**Correspondence to:**

Margaret Constanti

National Guideline Centre (NGC)

11 St Andrews Place, Regent’s Park, London NW1 4LE

[m.constanti@hotmail.co.uk](mailto:m.constanti@hotmail.co.uk)

+44 (0) 2037 457379 ext. 1005

Table of Contents

[Technical appendix 4](#__RefHeading___Toc47372968)

[Additional methodological details 5](#__RefHeading___Toc47372969)

[1. Model inputs 5](#__RefHeading___Toc47372970)

[2. Cardiovascular events 5](#__RefHeading___Toc47372971)

[3. CVD risk 5](#__RefHeading___Toc47372972)

[4. Adverse events 5](#__RefHeading___Toc47372973)

[5. QALY loss due to adverse events 6](#__RefHeading___Toc47372974)

[6. Resource use: Drug costs 6](#__RefHeading___Toc47372975)

[7. Hospital costs: 6](#__RefHeading___Toc47372976)

[8. Model Validation 7](#__RefHeading___Toc47372977)

[9. Sensitivity analyses: 7](#__RefHeading___Toc47372978)

[Additional Results 8](#__RefHeading___Toc47372979)

[10. Sensitivity analyses further details 8](#__RefHeading___Toc47372980)

[Supplementary tables and figures: 9](#__RefHeading___Toc47372981)

[Alternative treatment effects 14](#__RefHeading___Toc47372982)

[Additional results tables 16](#__RefHeading___Toc47372983)

[Figures…. 21](#__RefHeading___Toc47372984)

[References 24](#__RefHeading___Toc47372985)

Technical appendix

# Additional methodological details

## Model inputs

Model inputs were based on clinical evidence identified in a systematic review (see chapter C, NICE 2019 hypertension guideline),^1^ supplemented by additional data sources as required (see Table 1 & supplemental tables S1, S2, S3, S4, S5, S7, S8, S9, S10). Model inputs were validated with clinical members of the guideline committee.

## Cardiovascular events

The non-fatal cardiovascular events considered were: stable angina (SA); unstable angina (UA); myocardial infarction (MI); transient ischaemic attack (TIA); stroke; and heart failure (HF). Heart failure is not always included in risk calculators,^2^ but evidence showed that antihypertensive treatment reduces the risk of new heart failure.^3^

The relative distributions of first CVD events based on age and sex, other than heart failure, were directly extracted from the literature and are shown in table S1.^4^ The relative distribution for heart failure was calculated using the incidence of heart failure relative to the total incidence of the other CVD events for each age group and gender.^5^

## CVD risk

CVD risk was determined only by the pre-defined risk subgroup in the model. In particular, the starting age and gender of the population being modelled was independent of risk. For example, if the focus was on the 10% risk subgroup: whether the starting cohort was aged 40 or 70 did not affect the level of risk, as the CVD risk being modelled was still a risk of 10%. However, the distribution of events within that 10% risk varied by age and gender.^6^ Age subgroups were incorporated into the model because an event avoided at a younger age would accrue benefits over a longer period of time. Additionally, non-CVD mortality varied by age and gender.

## Adverse events

SPRINT reported injurious falls and acute kidney injury (AKI) resulting in hospitalisation (i.e. serious adverse events). These risks were applied to those aged 60 and over on treatment, reflecting the population in SPRINT.

The relative risk of AKI for those over 75 versus under 75 (in the standard treatment arm) was calculated from a SPRINT sub-study.^7^ and applied to the probability of AKI for those aged over 75.

## QALY loss due to adverse events

Disutility associated with AKI was based on that of renal failure (0.525), taken from the Sullivan catalogue of EQ-5D utilities at an average age of 60 years (rounded up to the nearest 10) and subtracted from the general population utility for that age.^8^

The disutility from a fall was based on a hip fracture, and taken from a systematic review on utilities associated with Osteoporosis.^9^

## Resource use: Drug costs

The most commonly prescribed drug in each class was extracted from Prescription Cost Analysis.^10^ No specific data for drug prescription was available for stage 1 hypertension and so general population prescriptions were used. Drug costs were applied to the percentage of people on 1, 2 or 3 plus drugs by age band and gender based on data from 27 GP practices from the CPRD database^11^ (personal communication S Ley-Flurrie) (Table S3).

Monitoring resource use and costs were based on the number of consultations needed by number of drugs, and the number of tests needed by type of drug (see Table S5). A UK study showed the average follow-up frequency after intensification of medication was about 1.3 months, and mean time from recording a raised BP to intensification of medication was around 6 months.^12^ The model was simplified by applying all first-year costs of the different steps of treatment in the first year of the model, a conservative assumption because some people may in fact die before treatment escalation. The average number of consultations when established on treatment based on CPRD data was 1.9 GP consultations per year.^13^ Blood pressure monitoring was assumed to happen during consultations.

## Hospital costs:

Where the source was stated as NHS reference costs, this included all resources related to the hospital admission. For stroke: The event state included NHS and social care costs. Recurrent strokes were also included in the costs. The costs for TIA, MI, unstable angina, and heart failure were all from the same source (See Table 1), and included all healthcare costs after a first event. See full model write-up for further detail.

Resource use associated with adverse events was based on the cost and length of hospital stays for AKI, or injurious falls (NICE CG161)^14^. The average length of hospital stay following a fall was reported as 2.7 days.^15^ A greater stay of 8.6 days for those admitted for a fall aged over 65 years (personal communication, Julia Titterton) was tested in a sensitivity analysis.

## Model Validation

Results were validated by comparing undiscounted life years for men and women age 60 on no treatment (taking a straight average of the life years from the four risk subgroups) with the life expectancy of men and women aged 60 from the Life tables for England 2016 (the source used for life expectancy in the model).^16^

## Sensitivity analyses:

Probabilistic Sensitivity Analysis (PSA)

PSA was undertaken to assess parameter uncertainty. Where possible, distributions were attached to inputs in the model. For the distribution of first events and of people on 1, 2 and 3 drugs, the dirichlet distribution was used. For the incidence of heart failure, probabilities of adverse events, and utilities, the beta distribution was used, which is bounded between 0 and 1. SMR’s and relative risks were made probabilistic using the lognormal distribution. The model was run for 5000 simulations for the base case and each probabilistic sensitivity analysis.

Deterministic sensitivity analyses (DSA)

DSA were conducted including running the model for alternative age groups (probabilistic) and testing differential treatment durations in the no treatment group (probabilistic), which involved modelling patients starting treatment after various defined periods of time (e.g. 5 years, see table S7), to capture that people may develop other risk factors over time that would make them eligible for treatment, as this was not captured explicitly in the model (apart from people going onto treatment if they had a CVD event).

Threshold Analysis

The minimum cardiovascular risk levels by sex and age group were calculated using the UK QRISK2 calculator,^17^ to assess whether the threshold risk levels identified by the model were clinically feasible using the following values:

- Untreated SBP of 140 mmHg for all age groups (minimum for stage 1 hypertension)
- Total cholesterol (TC): high-density lipoprotein cholesterol (HDL-C) ratio of 2.5. (estimated from 2.5^th^ percentile from the National Diet and Nutrition Survey)^18^
- All other variables within the calculator were left blank.

# Additional Results

## Sensitivity analyses further details

Treatment effect

Table S14 shows that using the upper confidence interval of treatment effect can lead to treatment being dominated in all groups. This is because some of the upper confidence intervals were above 1. The relative cardiovascular risks used in the base case were considered very conservative, and tables S12 and S13 support the inference that less conservative relative risks would make treatment even more cost effective, and these are likely to be closer to the treatment effect in practice.

Differential treatment duration

For men, the assumptions made about differential treatment duration affected the base-case conclusion in younger people, as there was some uncertainty about whether it was cost-effective to treat everyone in these groups if they may become eligible for treatment in a shorter time frame. For women, the differential treatment durations did not impact the base case conclusions because it was still not cost effective to treat all younger women (aged 40 and 50), regardless of durations tested.

References

1. National Institute for Health and Care Excellence. *Hypertension in Adults: Diagnosis and Management (NG136)*.; 2019.

2. ClinRisk Ltd. Qrisk 2-2017 risk calculator. Published online 2017.

3. Brunström M, Carlberg B. Association of blood pressure lowering with mortality and cardiovascular disease across blood pressure levels a systematic review and meta-analysis. *JAMA Intern Med*. 2018;178(1):28-36. doi:10.1001/jamainternmed.2017.6015

4. National Institute for Health and Clinical Excellence. *Statins for the Preventions of Cardiovascular Events (TA94)*.; 2006.

5. Cowie MR, Wood DA, Coats AJS, et al. Incidence and aetiology of heart failure: A population-based study. *Eur Heart J*. 1999;20(6):421-428. doi:10.1053/euhj.1998.1280

6. National Clinical Guideline Centre. *Cardiovascular Risk Assessment and Reduction, Including Lipid Modification (CG181)*. NICE; 2014.

7. Rocco M V., Sink KM, Lovato LC, et al. Effects of Intensive Blood Pressure Treatment on Acute Kidney Injury Events in the Systolic Blood Pressure Intervention Trial (SPRINT). *Am J Kidney Dis*. 2018;71(3):352-361. doi:10.1053/j.ajkd.2017.08.021

8. Sullivan PW, Slejko JF, Sculpher MJ, Ghushchyan V. Catalogue of EQ-5D scores for the United Kingdom. *Med Decis Mak*. 2011;31(6):800-804. doi:10.1177/0272989X11401031

9. Peasgood T, Herrmann K, Kanis JA, Brazier JE. An updated systematic review of health state utility values for osteoporosis related conditions. *Osteoporos Int*. 2009;20(6):853-868. doi:10.1007/s00198-009-0844-y

10. Prescription Cost Analysis - NHS Digital.

11. Clinical Practice Research Datalink | CPRD.

12. Xu W, Goldberg SI, Shubina M, Turchin A. Optimal systolic blood pressure target, time to intensification, and time to follow-up in treatment of hypertension: Population based retrospective cohort study. *BMJ*. 2015;350. doi:10.1136/bmj.h158

13. McManus RJ. Evaluating the impact of the 2011 NICE Hypertension Guideline on the Management of Hypertension in Primary Care and Subsequent Outcomes — SPCR.

14. National Institute for Health and Care Excellence. *Falls in Older People: Assessing Risk and Prevention (CG161)*.; 2013.

15. Kenny R., O’Shea D, Walker H. Impact of a Dedicated Syncope and Falls Facility for Older Adults on Emergency Beds - PubMed. *Age Ageing*. 2002;31(4):272-275.

16. National life tables, UK: 2014 to 2016 - Office for National Statistics.

17. Hippisley-Cox J, Coupland C, Vinogradova Y, et al. Predicting cardiovascular risk in England and Wales: Prospective derivation and validation of QRISK2. *BMJ*. 2008;336(7659):1475-1482. doi:10.1136/bmj.39609.449676.25

18. Public Health England FSA. *NDNS: Results from Years 7 and 8 (Combined): Results of the National Diet and Nutrition Survey (Ndns) Rolling Programme for 2014 to 2015 and 2015 to 2016*.; 2018. https://www.gov.uk/government/statistics/ndns-results-from-years-7-and-8-combined

Supplementary tables and figures:

Table S1: Relative distribution of CVD events including heart failure

| Age | SA | UA | MI | TIA | Stroke | HF | CVD death | Total CVD risk (b) |
| --- | --- | --- | --- | --- | --- | --- | --- | --- |
| **Male** | | | | | | | | |
| 40-44 | 30.7% | 10.7% | 29.5% | 6.0% | 12.9% | 7.1% | 10.1% | 107.1% |
| 45-54 | 30.7% | 10.7% | 29.5% | 6.0% | 12.9% | 7.1% | 10.1% | 107.1% |
| 55-64 | 32.8% | 7.1% | 17.2% | 8.9% | 20.6% | 12.4% | 13.4% | 112.4% |
| 65-74 | 21.4% | 8.3% | 17.3% | 10.0% | 27.0% | 16.0% | 16.0% | 116.0% |
| 75-84 | 19.1% | 8.1% | 16.1% | 8.0% | 34.3% | 26.1% | 14.3% | 126.1% |
| **Female** | | | | | | | | |
| 40-44 | 32.4% | 11.7% | 8.0% | 16.0% | 22.9% | 6.3% | 9.1% | 106.3% |
| 45-54 | 32.4% | 11.7% | 8.0% | 16.0% | 22.9% | 6.3% | 9.1% | 106.3% |
| 55-64 | 34.6% | 7.3% | 9.2% | 9.5% | 28.8% | 10.6% | 10.6% | 110.6% |
| 65-74 | 20.2% | 5.2% | 12.1% | 7.3% | 38.2% | 18.5% | 17.1% | 118.5% |
| 75-84 | 14.9% | 3.4% | 10.2% | 9.8% | 46.4% | 25.2% | 15.2% | 125.2% |

1. There was no data for age below 45 and so the age 40 subgroup (35-44 age range) data is the same as the age 50 subgroup data (45-54 age range).
2. The total CVD risk sums the distribution of all columns (that is, events) in the table, so this also includes heart failure, which is not typically included in risk calculators and therefore not included in the risk subgroups being modelled (5%, 10%, 15%, 20%).

Table S2: Base case relative risks of CVD events and CVD death

|  | 35-44 | 45-54 | 55-64 | 65-74 | 75+ |
| --- | --- | --- | --- | --- | --- |
| **CHD events** | | | | | |
| Men | 0.86 | 0.84 | 0.86 | 0.91 | 0.90 |
| Women | 0.84 | 0.84 | 0.86 | 0.90 | 0.89 |
| **Stroke events** | | | | | |
| Men | 0.84 | 0.83 | 0.86 | 0.93 | 0.92 |
| Women | 0.81 | 0.82 | 0.86 | 0.92 | 0.90 |
| **Heart failure events** | | | | | |
| Men | 0.85 | 0.84 | 0.87 | 0.94 | 0.94 |
| Women | 0.82 | 0.83 | 0.87 | 0.93 | 0.91 |
| **Cardiovascular mortality** | | | | | |
| Men | 0.84 | 0.83 | 0.86 | 0.93 | 0.92 |
| Women | 0.81 | 0.82 | 0.86 | 0.92 | 0.90 |

The CHD relative risk was applied to the MI, stable angina and unstable angina health states. The stroke relative risk was applied to the stroke and TIA health states. The heart failure relative risk was applied to the heart failure health state. The cardiovascular mortality relative risk was applied to the CV death state.

Table S3: Proportion of patients on different numbers of drug by age

| Age | Men | | | Women | | |
| --- | --- | --- | --- | --- | --- | --- |
|  | 1 | 2 | 3+ | 1 | 2 | 3+ |
| 35-44 | 61% | 31% | 8% | 62% | 28% | 11% |
| 45-54 | 53% | 33% | 14% | 58% | 32% | 10% |
| 55-64 | 44% | 38% | 18% | 51% | 35% | 13% |
| 65-74 | 39% | 39% | 22% | 44% | 38% | 18% |
| 75+ | 38% | 40% | 22% | 41% | 39% | 20% |

Table S4: Age adjustments applied to relative treatment effect in model

|  | 35–44 | 45–54 | 55–64 (reference) | 65–74 | 75+ |
| --- | --- | --- | --- | --- | --- |
| **CHD events** | | | | | |
| Men | 1.00 | 0.98 | 1.00 | 1.06 | 1.05 |
| Women | 0.97 | 0.98 | 1.00 | 1.05 | 1.03 |
| **Stroke events (a)** | | | | | |
| Men | 0.98 | 0.96 | 1.00 | 1.08 | 1.07 |
| Women | 0.94 | 0.96 | 1.00 | 1.07 | 1.05 |

1. There was no data on relative risk for heart failure by age from the Law meta-analysis, therefore the age adjustments for stroke were applied to the heart failure treatment effect data from the clinical review. The stroke adjustments were also applied to the cardiovascular death relative risk

Note: The 55–64 age group is the reference group. The 65–74 and 75-and-older age subgroups both use the relative risks from the 70–79 age group in the Law meta-analysis to derive the age adjustments. There were treatment effects reported in Law for an 80–89 year old age group also, but these were not used to apply age adjustments to a group older than 75, as there was a trend of increasing relative risks in older age in the Law data. The Brunström relative risks were already felt to be conservative. Note also that anyone on treatment surviving to aged older than 75 will be applied the age 75 age group treatment effect. If the relationship between age and relative risks is to be believed from Law, this means that the older someone is, the less they benefit from treatment. By not applying smaller relative risks to people aged over 75, this means that we may have been modelling treatment as being more effective than it might be. However, the base-case treatment effects are very conservative anyway, so these effects on the model are likely to balance out.

Table S5: Monitoring resource use

| YEAR 1 | | | | |
| --- | --- | --- | --- | --- |
|  | No treatment | 1 drug | 2 drugs | 3 drugs |
| Number of consultations | 1 | 2 | 3 | 4 |
|  |  |  |  |  |
| Tests | **No treatment** | **A drugs** | **C drugs** | **D drugs** |
| Clinical biochemistry (renal panel) | 0 | 4 | 1 | 2 |
| Albumin: creatinine ratio | 0 | 1 | 0 | 1 |
| **SUBSEQUENT YEARS** | | | | |
|  | **No treatment** | **All drugs** | | |
| Number of consultations | 1 | 1.87 | | |
| Tests |  |  | | |
| Clinical biochemistry | 0 | 1 | | |
| Albumin: creatinine ratio | 0 | 0.2 | | |

Note: A drugs = ACE/ARB, C drugs = CCB, D drugs = diuretic.

Table S6: 10 year numbers needed to treat

| Age | Minimum cardiovascular risk level | absolute risk reduction | NNTs | Interpretation | | |
| --- | --- | --- | --- | --- | --- | --- |
| **10 YEAR NNT’S** | | | | | | |
| **Male** |  |  |  |  | | |
| 40 | 1.50% | 0.013 | 79 | need to treat | 79 | men to avoid 1 event |
| 50 | 4.00% | 0.033 | 30 | need to treat | 30 | men to avoid 1 event |
| 60 | 8.50% | 0.073 | 14 | need to treat | 14 | men to avoid 1 event |
| 70 | 16.40% | 0.152 | 7 | need to treat | 7 | men to avoid 1 event |
| 75 | 22% | 0.206 | 5 | need to treat | 5 | men to avoid 1 event |
| **Female** |  |  |  |  | | |
| 40 | 0.90% | 0.007 | 136 | need to treat | 136 | women to avoid 1 event |
| 50 | 2.30% | 0.019 | 52 | need to treat | 52 | women to avoid 1 event |
| 60 | 5.30% | 0.046 | 22 | need to treat | 22 | women to avoid 1 event |
| 70 | 11.70% | 0.107 | 9 | need to treat | 9 | women to avoid 1 event |
| 75 | 17.00% | 0.153 | 7 | need to treat | 7 | women to avoid 1 event |

Table S7: Differential treatment durations tested by age group

| Age subgroup | Durations of differential treatment tested |
| --- | --- |
| 40, 50 | 1, 5, 10 and 20 years |
| 60 | 1, 5 and 10 years |
| 70, 75 | 1 and 5 years |

### Alternative treatment effects

Table S8: Relative risk of CHD and stroke events with antihypertensive treatment using Law 2009

|  | 35-44 | 45-54 | 55-64 | 65-74 | 75+ |
| --- | --- | --- | --- | --- | --- |
| **CHD events** | | | | | |
| Men | 0.65 | 0.64 | 0.65 | 0.69 | 0.69 |
| Women | 0.65 | 0.65 | 0.67 | 0.70 | 0.69 |
| **Stroke events** | | | | | |
| Men | 0.55 | 0.54 | 0.56 | 0.61 | 0.60 |
| Women | 0.55 | 0.56 | 0.58 | 0.62 | 0.61 |
| **Heart failure events** | | | | | |
| Men | 0.67 | 0.66 | 0.63 | 0.62 | 0.62 |
| Women | 0.68 | 0.67 | 0.64 | 0.63 | 0.62 |
| **Cardiovascular mortality** | | | | | |
| Men | 0.62 | 0.61 | 0.62 | 0.66 | 0.64 |
| Women | 0.59 | 0.60 | 0.61 | 0.66 | 0.63 |

1. The RRs from the meta-analysis were taken from the following age groups: for the 35**–**44 age subgroup, the age 40**–**49 RRs were used; for the 45**–**54 age subgroup, the 50**–**59 RRs were used; for the 55**–**64 age subgroup, the 60**–**69 RRs were used; for the 65**–**74 and 75 age subgroups, the 70**–**79 RRs were used.

Table S9: Estimated and proportional SBP reduction based on number of drugs (Law 2009)

| Pre-treatment systolic BP | No. of drugs | Estimated reduction in systolic BP (a) | Proportional systolic BP reduction in reference to 1 drug (b) |
| --- | --- | --- | --- |
| 150 | 1 | 8.7 |  |
| 150 | 2 | 16.5 | 1.90 |
| 150 | 3 | 23.6 | 2.71 |

1. Taken from table 3, Law 2009.
2. Calculated.

Table S10: Dose adjusted Brunström relative risks, by age

|  | 35-44 | 45-54 | 55-64 | 65-74 | 75+ |
| --- | --- | --- | --- | --- | --- |
| **CHD events** | | | | | |
| Men | 0.81 | 0.80 | 0.78 | 0.77 | 0.77 |
| Women | 0.81 | 0.81 | 0.80 | 0.78 | 0.78 |
| **Stroke events** | | | | | |
| Men | 0.81 | 0.80 | 0.78 | 0.77 | 0.77 |
| Women | 0.81 | 0.81 | 0.80 | 0.78 | 0.78 |
| **Heart failure events** | | | | | |
| Men | 0.82 | 0.81 | 0.80 | 0.79 | 0.79 |
| Women | 0.82 | 0.82 | 0.81 | 0.80 | 0.79 |
| **Cardiovascular mortality** | | | | | |
| Men | 0.81 | 0.80 | 0.78 | 0.77 | 0.77 |
| Women | 0.81 | 0.81 | 0.80 | 0.78 | 0.78 |

### Additional results tables

Table S11: Differential treatment duration results for all ages

| Years before meeting other criteria for treatment | Risk threshold | | | | | | | | |
| --- | --- | --- | --- | --- | --- | --- | --- | --- | --- |
|  | Age 40 | Age 50 | | Age 60 | | Age 70 | | Age 75 | |
| MALES |  |  | |  | |  | |  | |
| 1 | 4.2% | 4.1% | | 6.3% | | 10.6% | | 11.8% | |
| 5 | 3.5% | 3.5% | | 5.6% | | 10.3% | | 11.6% | |
| 10 | 2.6% | 2.7% | | 4.8% | | - | | - | |
| 20 | 1.3% | 1.9% | | - | | - | | - | |
| ***Never (base case)*** | **0.7%** | **1.8%** | | **5.0%** | | **9.7%** | | **11.4%** | |
| **Minimum cardiovascular risk level in population** | 1.5% | 4.0% | | 8.5% | | 16.4% | | 22.3% | |
| FEMALES |  |  |  | |  | |  | |  |
| 1 | 2.6% | 2.8% | 4.8% | | 7.5% | | 8.1% | |  |
| 5 | 2.3% | 2.6% | 4.6% | | 7.6% | | 8.1% | |  |
| 10 | 2.0% | 2.3% | 4.5% | | - | | - | |  |
| 20 | 1.6% | 2.6% | - | | - | | - | |  |
| ***Never (base case)*** | **1.7%** | **2.8%** | **4.9%** | | **7.5%** | | **8.5%** | |  |
| **Minimum cardiovascular risk level in population** | 0.9% | 2.3% | 5.3% | | 11.7% | | 17.0% | |  |

The columns show the risk thresholds for the different age groups. The rows show the differential treatment durations tested and the results of the base-case analysis for each age group (that is, where a lifetime of treatment was compared to a lifetime of no treatment – except if people had a CVD event). Additionally, the minimum cardiovascular risk values in the population for each age and gender are also presented. Cells that are orange indicate that the risk thresholds for that age are below the minimum cardiovascular risk values for that age in the population. If this is the case, then this means that it is cost effective to treat all of that age and gender.

#### SA1: Using relative risks from Law 2009 (probabilistic)

Table S12: Using relative risks from Law 2009

| Analysis | Risk | Incremental cost | Incremental QALYs | ICER | Probability Tx CE at 20k | | Incremental cost | Incremental QALYs | ICER | Probability Tx CE at 20k | |
| --- | --- | --- | --- | --- | --- | --- | --- | --- | --- | --- | --- |
|  |  | Male | | | | Female | | | | |  |
| Age 60 (base case age) | 5% | £458 | 0.247 | £1,859 | 100% | | £387 | 0.213 | £1,821 | 100% | |
|  | 10% | -£27 | 0.374 | Dominant | 100% | | -£321 | 0.350 | Dominant | 100% | |
|  | 15% | -£452 | 0.483 | Dominant | 100% | | -£930 | 0.464 | Dominant | 100% | |
|  | 20% | -£821 | 0.574 | Dominant | 100% | | -£1,449 | 0.558 | Dominant | 100% | |
| Age 40 | 5% | -£128 | 0.404 | Dominant | 100% | | -£265 | 0.319 | Dominant | 100% | |
|  | 10% | -£728 | 0.548 | Dominant | 100% | | -£1,275 | 0.490 | Dominant | 100% | |
|  | 15% | -£1,225 | 0.660 | Dominant | 100% | | -£2,102 | 0.622 | Dominant | 100% | |
|  | 20% | -£1,635 | 0.745 | Dominant | 100% | | -£2,774 | 0.723 | Dominant | 100% | |
| Age 50 | 5% | £142 | 0.340 | £418 | 100% | | £67 | 0.271 | £249 | 100% | |
|  | 10% | -£391 | 0.480 | Dominant | 100% | | -£777 | 0.425 | Dominant | 100% | |
|  | 15% | -£836 | 0.595 | Dominant | 100% | | -£1,480 | 0.549 | Dominant | 100% | |
|  | 20% | -£1,212 | 0.686 | Dominant | 100% | | -£2,069 | 0.645 | Dominant | 100% | |
| Age 70 | 5% | £564 | 0.142 | £3,981 | 100% | | £479 | 0.144 | £3,333 | 100% | |
|  | 10% | £186 | 0.237 | £787 | 100% | | -£67 | 0.252 | Dominant | 100% | |
|  | 15% | -£153 | 0.322 | Dominant | 100% | | -£554 | 0.348 | Dominant | 100% | |
|  | 20% | -£458 | 0.397 | Dominant | 100% | | -£984 | 0.431 | Dominant | 100% | |
| Age 75 | 5% | £589 | 0.096 | £6,163 | 100% | | £518 | 0.105 | £4,914 | 100% | |
|  | 10% | £268 | 0.175 | £1,534 | 100% | | £59 | 0.195 | £303 | 100% | |
|  | 15% | -£28 | 0.247 | Dominant | 100% | | -£360 | 0.275 | Dominant | 100% | |
|  | 20% | -£301 | 0.312 | Dominant | 100% | | -£739 | 0.348 | Dominant | 100% | |

Cells shaded green mean treatment is a dominant intervention (both more effective and less expensive).

Abbreviations: CE = cost effective, 20k = £20,000, ICER = incremental cost effectiveness ratio, QALYS = quality adjusted life-years, Tx = treatment.

#### SA2: Adjusted base case data (Brunström) to take into account more medication (probabilistic)

Table S13 : Using adjusted Brunström relative risks taking into account more medication

| Analysis | Risk | Incremental cost | Incremental QALYs | ICER | Probability Tx CE at 20k | Incremental cost | | Incremental QALYs | ICER | Probability Tx CE at 20k | |
| --- | --- | --- | --- | --- | --- | --- | --- | --- | --- | --- | --- |
|  |  | Male | | | | | Female | | | |  |
| Age 60 (base case age) | 5% | £891 | 0.129 | £6,891 | 95% | £881 | | 0.110 | £7,998 | 92% | |
|  | 10% | £592 | 0.203 | £2,919 | 99% | £485 | | 0.184 | £2,633 | 98% | |
|  | 15% | £345 | 0.262 | £1,317 | 99% | £142 | | 0.243 | £584 | 99% | |
|  | 20% | £132 | 0.309 | £426 | 99% | -£144 | | 0.293 | Dominant | 100% | |
| Age 40 | 5% | £640 | 0.197 | £3,255 | 98% | £628 | | 0.152 | £4,132 | 96% | |
|  | 10% | £332 | 0.266 | £1,248 | 99% | £136 | | 0.231 | £589 | 99% | |
|  | 15% | £67 | 0.319 | £209 | 99% | -£261 | | £0 | Dominant | 99% | |
|  | 20% | -£140 | 0.356 | Dominant | 100% | -£562 | | 0.331 | Dominant | 100% | |
| Age 50 | 5% | £751 | 0.172 | £4,361 | 97% | £751 | | 0.133 | £5,631 | 95% | |
|  | 10% | £453 | 0.245 | £1,845 | 99% | £310 | | 0.209 | £1,478 | 99% | |
|  | 15% | £195 | 0.302 | £646 | 99% | -£61 | | £0 | Dominant | 100% | |
|  | 20% | £13 | 0.347 | £38 | 100% | -£346 | | 0.314 | Dominant | 99% | |
| Age 70 | 5% | £801 | 0.079 | £10,106 | 91% | £791 | | 0.079 | £10,066 | 89% | |
|  | 10% | £570 | 0.136 | £4,183 | 99% | £452 | | 0.143 | £3,156 | 98% | |
|  | 15% | £351 | 0.188 | £1,873 | 99% | £171 | | 0.195 | £879 | 99% | |
|  | 20% | £161 | 0.232 | £694 | 100% | -£96 | | 0.242 | Dominant | 99% | |
| Age 75 | 5% | £748 | 0.055 | £13,696 | 83% | £747 | | 0.058 | £12,885 | 82% | |
|  | 10% | £551 | 0.102 | £5,417 | 98% | £477 | | 0.109 | £4,385 | 97% | |
|  | 15% | £362 | 0.145 | £2,504 | 100% | £224 | | 0.155 | £1,445 | 99% | |
|  | 20% | £194 | 0.182 | £1,066 | 100% | -£5 | | 0.195 | Dominant | 99% | |

Cells shaded green mean treatment is a dominant intervention (both more effective and less expensive).

Abbreviations: CE = cost effective, 20k = £20,000, ICER = incremental cost effectiveness ratio, QALYS = quality adjusted life-years, Tx = treatment.

Table S14: Sensitivity analysis results (deterministic)

| Analysis | Risk | Incremental cost | Incremental QALYs | ICER | Incremental cost | Incremental QALYs | ICER |
| --- | --- | --- | --- | --- | --- | --- | --- |
|  |  | Male | | | Female | | |
| *Base case* | *5%* | £1,125 | 0.06 | £20,063 | £1,116 | 0.06 | £19,757 |
|  | *10%* | £932 | 0.10 | £9,807 | £846 | 0.10 | £8,572 |
|  | *15%* | £762 | 0.13 | £5,959 | £615 | 0.13 | £4,618 |
|  | *20%* | £613 | 0.16 | £3,948 | £418 | 0.16 | £2,601 |
| SA4: Lower CI of Base case treatment effect | 5% | £833 | 0.16 | £5,199 | £764 | 0.15 | £5,211 |
|  | 10% | £512 | 0.25 | £2,059 | £284 | 0.24 | £1,168 |
|  | 15% | £234 | 0.32 | £724 | -£122 | 0.32 | Dominant |
|  | 20% | -£4 | 0.39 | Dominant | -£462 | 0.39 | Dominant |
| SA5: Upper CI of Base case treatment effect | 5% | £1,421 | -0.06 | Dominated | £1,479 | -0.05 | Dominated |
|  | 10% | £1,351 | -0.08 | Dominated | £1,415 | -0.06 | Dominated |
|  | 15% | £1,278 | -0.09 | Dominated | £1,347 | -0.08 | Dominated |
|  | 20% | £1,205 | -0.10 | Dominated | £1,275 | -0.09 | Dominated |
| SA6: Annual CV risk increase for women the same as men | 5% | £1,125 | 0.06 | £20,063 | £1,006 | 0.07 | £14,350 |
|  | 10% | £932 | 0.10 | £9,807 | £774 | 0.11 | £7,327 |
|  | 15% | £762 | 0.13 | £5,959 | £573 | 0.13 | £4,243 |
|  | 20% | £613 | 0.16 | £3,948 | £398 | 0.16 | £2,511 |
| SA7: Annual CV risk increase for women halfway between women and men | 5% | £1,125 | 0.06 | £20,063 | £1,055 | 0.06 | £16,409 |
|  | 10% | £932 | 0.10 | £9,807 | £806 | 0.10 | £7,834 |
|  | 15% | £762 | 0.13 | £5,959 | £591 | 0.13 | £4,393 |
|  | 20% | £613 | 0.16 | £3,948 | £406 | 0.16 | £2,540 |
| SA8: Lower drug costs by 50% | 5% | £983 | 0.06 | £17,532 | £972 | 0.06 | £17,208 |
|  | 10% | £799 | 0.10 | £8,408 | £712 | 0.10 | £7,214 |
|  | 15% | £638 | 0.13 | £4,986 | £490 | 0.13 | £3,681 |
|  | 20% | £496 | 0.16 | £3,198 | £302 | 0.16 | £1,878 |
| SA9: Increase drug costs by 50% | 5% | £1,267 | 0.06 | £22,593 | £1,260 | 0.06 | £22,307 |
|  | 10% | £1,065 | 0.10 | £11,206 | £980 | 0.10 | £9,931 |
|  | 15% | £886 | 0.13 | £6,932 | £740 | 0.13 | £5,556 |
|  | 20% | £729 | 0.16 | £4,698 | £534 | 0.16 | £3,323 |
| SA10: Half health state costs | 5% | £1,214 | 0.06 | £21,646 | £1,246 | 0.06 | £22,073 |
|  | 10% | £1,076 | 0.10 | £11,322 | £1,065 | 0.10 | £10,787 |
|  | 15% | £952 | 0.13 | £7,444 | £905 | 0.13 | £6,796 |
|  | 20% | £840 | 0.16 | £5,414 | £764 | 0.16 | £4,759 |
| SA11: Double health state costs | 5% | £947 | 0.06 | £16,896 | £854 | 0.06 | £15,127 |
|  | 10% | £644 | 0.10 | £6,777 | £409 | 0.10 | £4,144 |
|  | 15% | £382 | 0.13 | £2,989 | £35 | 0.13 | £263 |
|  | 20% | £158 | 0.16 | £1,016 | -£276 | 0.16 | Dominant |
| SA12: Nurse doing appointment instead of GP | 5% | £759 | 0.06 | £13,532 | £727 | 0.06 | £12,877 |
|  | 10% | £588 | 0.10 | £6,185 | £483 | 0.10 | £4,891 |
|  | 15% | £439 | 0.13 | £3,431 | £275 | 0.13 | £2,068 |
|  | 20% | £309 | 0.16 | £1,994 | £101 | 0.16 | £627 |
| SA13: No. of consultations for first yr on treatment being doubled | 5% | £1,213 | 0.06 | £21,630 | £1,202 | 0.06 | £21,282 |
|  | 10% | £1,013 | 0.10 | £10,661 | £925 | 0.10 | £9,371 |
|  | 15% | £837 | 0.13 | £6,545 | £687 | 0.13 | £5,160 |
|  | 20% | £682 | 0.16 | £4,395 | £484 | 0.16 | £3,013 |
| SA14: Having no adverse events | 5% | £704 | 0.06 | £11,557 | £659 | 0.06 | £10,681 |
|  | 10% | £539 | 0.10 | £5,416 | £422 | 0.10 | £4,079 |
|  | 15% | £396 | 0.13 | £2,996 | £222 | 0.14 | £1,613 |
|  | 20% | £272 | 0.16 | £1,707 | £54 | 0.16 | £327 |
| SA15: Longer length of stay for falls | 5% | £1,623 | 0.06 | £28,953 | £1,650 | 0.06 | £29,217 |
|  | 10% | £1,399 | 0.10 | £14,727 | £1,345 | 0.10 | £13,626 |
|  | 15% | £1,200 | 0.13 | £9,386 | £1,080 | 0.13 | £8,113 |
|  | 20% | £1,023 | 0.16 | £6,592 | £851 | 0.16 | £5,301 |
| SA16: Apply over 75s AKI risk to falls also | 5% | £1,227 | 0.05 | £22,328 | £1,241 | 0.06 | £22,508 |
|  | 10% | £1,022 | 0.09 | £10,861 | £955 | 0.10 | £9,795 |
|  | 15% | £840 | 0.13 | £6,613 | £710 | 0.13 | £5,370 |
|  | 20% | £680 | 0.15 | £4,404 | £499 | 0.16 | £3,125 |
| SA17: Apply fall utility loss for 4 months | 5% | £1,125 | 0.05 | £23,995 | £1,116 | 0.05 | £23,930 |
|  | 10% | £932 | 0.09 | £10,785 | £846 | 0.09 | £9,453 |
|  | 15% | £762 | 0.12 | £6,361 | £615 | 0.12 | £4,936 |
|  | 20% | £613 | 0.15 | £4,150 | £418 | 0.15 | £2,737 |
| SA18: Utilities lower CI | 5% | £1,125 | 0.06 | £18,718 | £1,116 | 0.06 | £18,215 |
|  | 10% | £932 | 0.10 | £9,136 | £846 | 0.11 | £7,891 |
|  | 15% | £762 | 0.14 | £5,543 | £615 | 0.14 | £4,243 |
|  | 20% | £613 | 0.17 | £3,667 | £418 | 0.18 | £2,384 |
| SA19: Utilities upper CI | 5% | £1,125 | 0.05 | £21,615 | £1,116 | 0.05 | £21,585 |
|  | 10% | £932 | 0.09 | £10,585 | £846 | 0.09 | £9,382 |
|  | 15% | £762 | 0.12 | £6,442 | £615 | 0.12 | £5,066 |
|  | 20% | £613 | 0.14 | £4,275 | £418 | 0.15 | £2,861 |
| SA20: Double SMR for HF | 5% | £1,127 | 0.06 | £19,607 | £1,118 | 0.06 | £19,278 |
|  | 10% | £935 | 0.10 | £9,612 | £850 | 0.10 | £8,404 |
|  | 15% | £766 | 0.13 | £5,858 | £620 | 0.14 | £4,549 |
|  | 20% | £618 | 0.16 | £3,895 | £423 | 0.16 | £2,579 |

Note that cells shaded red are above the NICE cost effectiveness threshold of £20,000 per QALY. Dominated means an intervention is both more expensive and less effective than the comparator. Cells shaded green mean treatment is a dominant intervention (both more effective and less expensive).

*The base-case results presented in the table below for reference are the deterministic results.*

Abbreviations: CE = cost effective, 20k = £20,000, ICER = incremental cost effectiveness ratio, QALYS = quality adjusted life-years.

### Figures


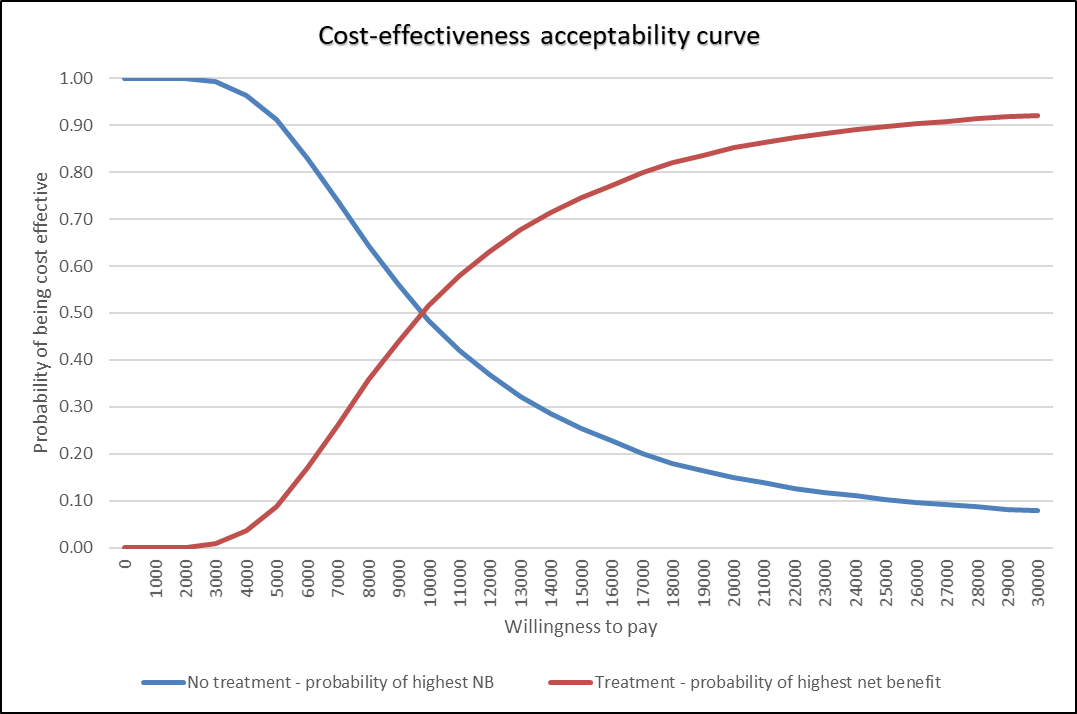


**Figure S1: Cost effectiveness acceptability curve – males, age 60, 10% risk**


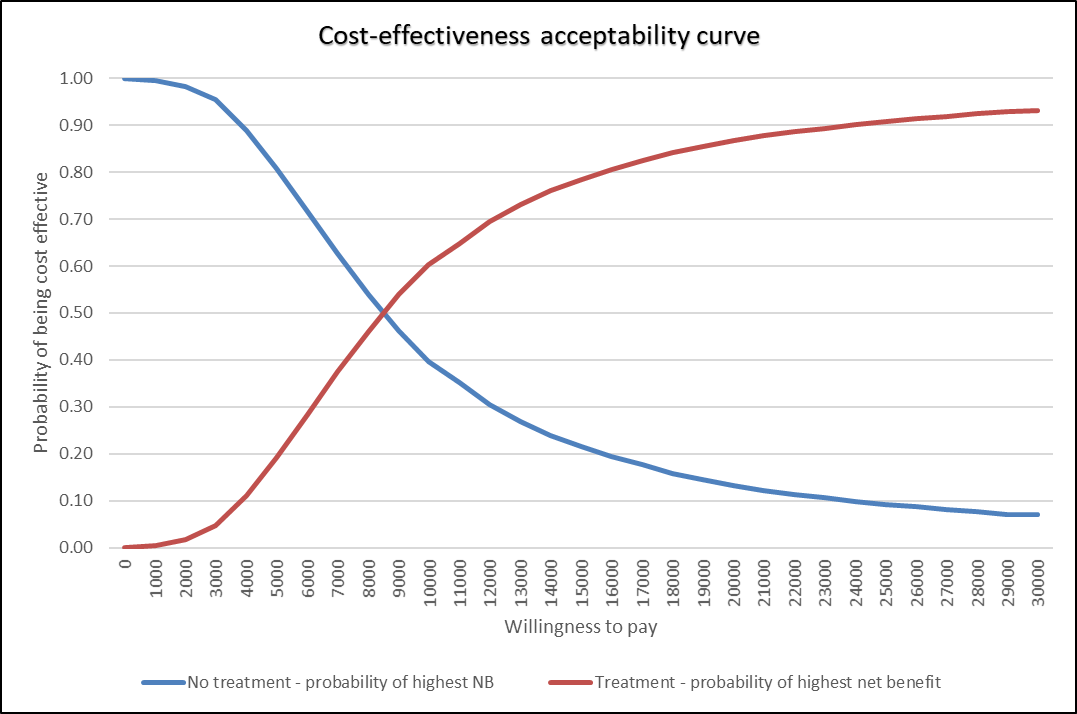


**Figure S2: Cost effectiveness acceptability curve – females, age 60, 10% risk**


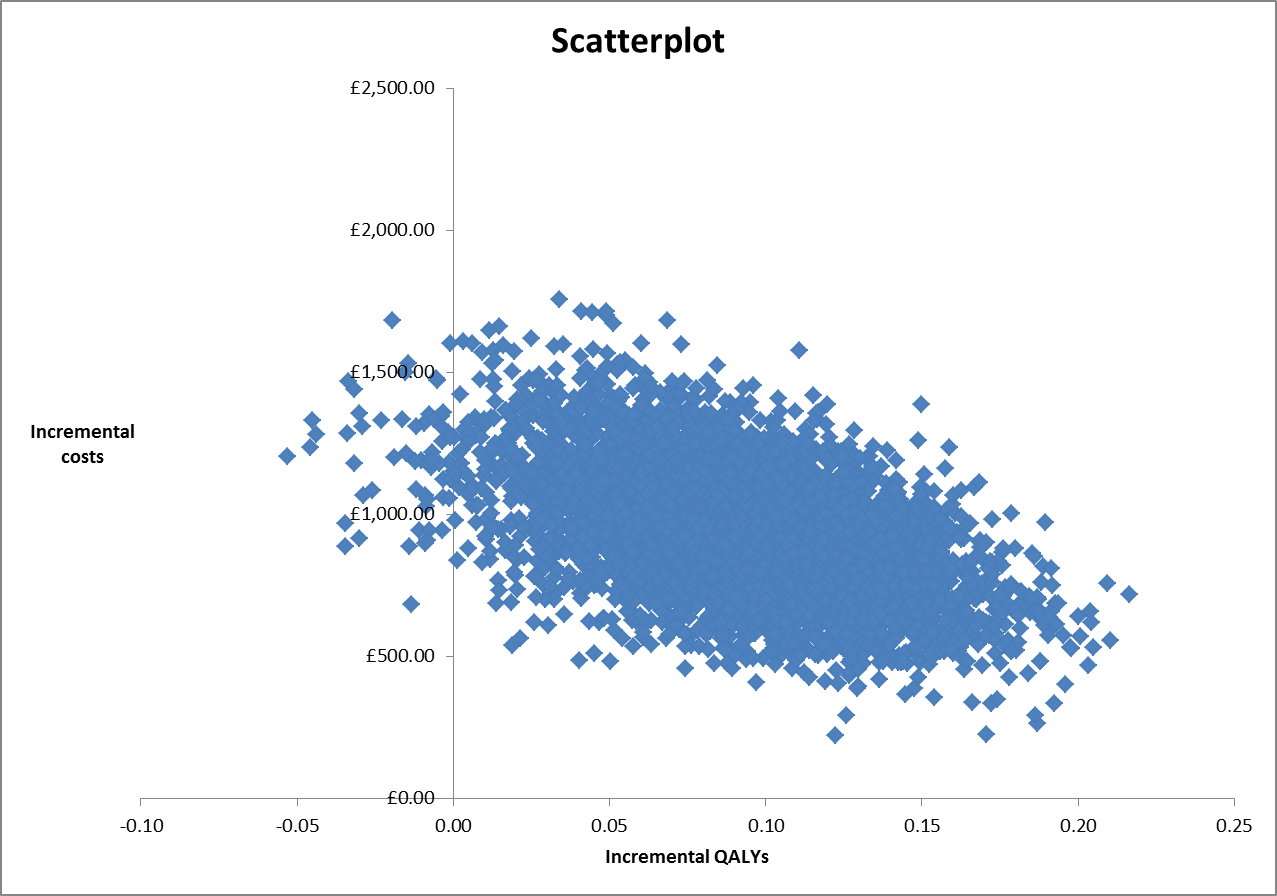


**Figure S3: Scatterplot – males, age 60, 10% risk**


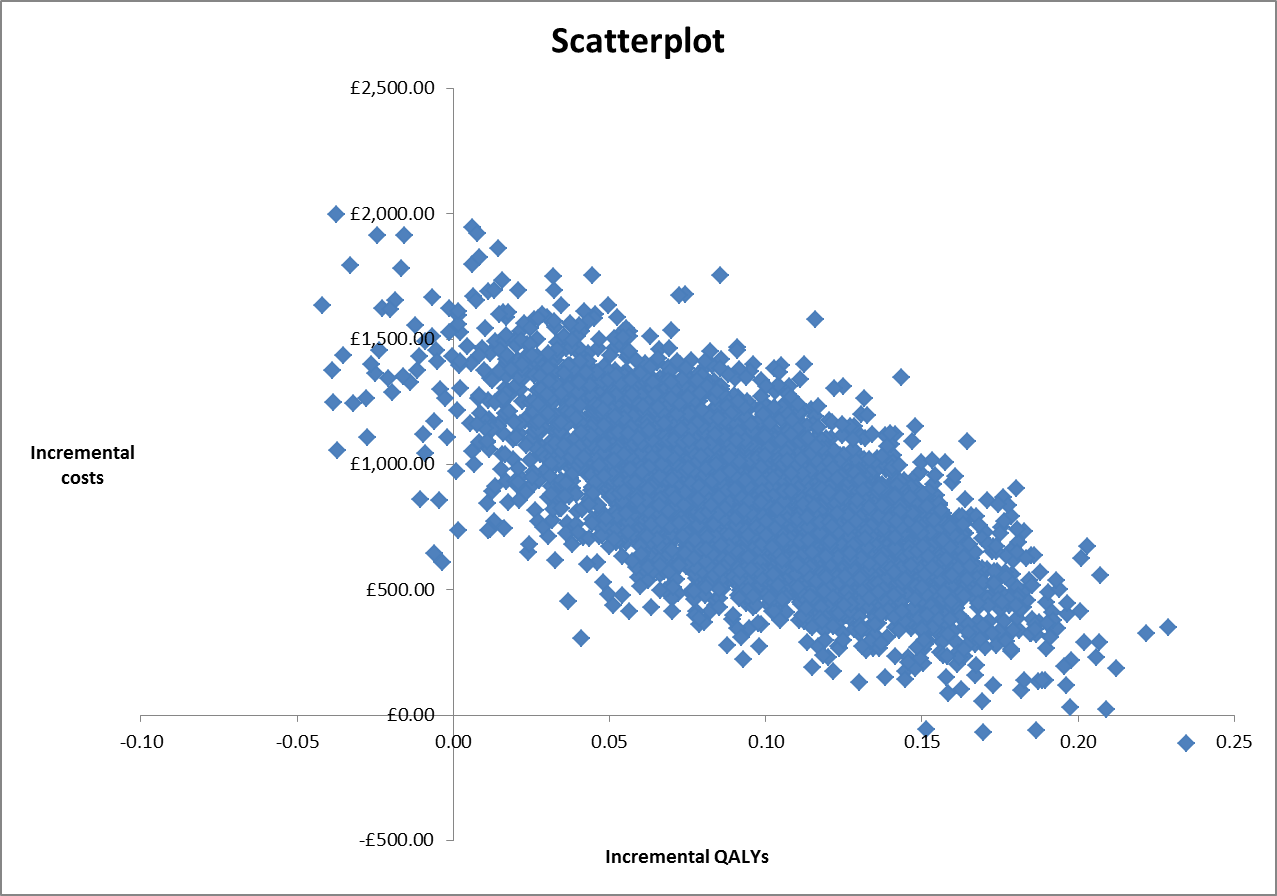


**Figure S4: Scatterplot – females, age 60, 10% risk**
